# Alterations in Leg Extensor Muscle-Tendon Unit Biomechanical Properties With Ageing and Mechanical Loading

**DOI:** 10.3389/fphys.2018.00150

**Published:** 2018-02-28

**Authors:** Christopher McCrum, Pamela Leow, Gaspar Epro, Matthias König, Kenneth Meijer, Kiros Karamanidis

**Affiliations:** ^1^Department of Human Movement Science, NUTRIM School of Nutrition and Translational Research in Metabolism, Maastricht University Medical Centre+, Maastricht, Netherlands; ^2^Institute of Movement and Sport Gerontology, German Sport University Cologne, Cologne, Germany; ^3^Sport and Exercise Science Research Centre, School of Applied Sciences, London South Bank University, London, United Kingdom

**Keywords:** Achilles tendon, aged, bed rest, locomotion, quadriceps femoris, patellar tendon, resistance training, triceps surae

## Abstract

Tendons transfer forces produced by muscle to the skeletal system and can therefore have a large influence on movement effectiveness and safety. Tendons are mechanosensitive, meaning that they adapt their material, morphological and hence their mechanical properties in response to mechanical loading. Therefore, unloading due to immobilization or inactivity could lead to changes in tendon mechanical properties. Additionally, ageing may influence tendon biomechanical properties directly, as a result of biological changes in the tendon, and indirectly, due to reduced muscle strength and physical activity. This review aimed to examine age-related differences in human leg extensor (triceps surae and quadriceps femoris) muscle-tendon unit biomechanical properties. Additionally, this review aimed to assess if, and to what extent mechanical loading interventions could counteract these changes in older adults. There appear to be consistent reductions in human triceps surae and quadriceps femoris muscle strength, accompanied by similar reductions in tendon stiffness and elastic modulus with ageing, whereas the effect on tendon cross sectional area is unclear. Therefore, the observed age-related changes in tendon stiffness are predominantly due to changes in tendon material rather than size with age. However, human tendons appear to retain their mechanosensitivity with age, as intervention studies report alterations in tendon biomechanical properties in older adults of similar magnitudes to younger adults over 12–14 weeks of training. Interventions should implement tendon strains corresponding to high mechanical loads (i.e., 80–90% MVC) with repetitive loading for up to 3–4 months to successfully counteract age-related changes in leg extensor muscle-tendon unit biomechanical properties.

## Introduction

The leg extensor muscle-tendon units (MTUs) play important roles in locomotion, with the muscles opposing gravity and controlling and generating progression by decelerating and accelerating the center of mass and the tendons storing and returning elastic energy to the musculoskeletal system (Biewener and Roberts, 2000; Roberts, 2002; Pandy and Andriacchi, 2010). As a consequence, the tendons can have a large influence on movement effectiveness (Hof et al., 2002; Lichtwark and Wilson, 2007; Pandy and Andriacchi, 2010; Huang et al., 2015). Specifically, the mechanical properties of the Achilles (AT) and patellar (PT) tendons (e.g., tendon stiffness) can greatly influence the contributions of the triceps surae (TS) and quadriceps femoris (QF) to forward propulsion and energy absorption during gait.

In the literature, it is well established that ageing mammalian tendons experience biochemical, cellular, mechanical and pathological alterations, causing progressive deterioration (Noyes and Grood, 1976; Vogel, 1991; Kjaer, 2004; Komatsu et al., 2004). *In vitro*, the connective tissues of older adults have a declined failure stress compared to younger adults (Noyes and Grood, 1976). *In vitro* animal studies have associated ageing with an increase in irreducible collagen cross-linking, a reduction in collagen fibril diameter and its crimp angle, an increase in more extensible elastin content, a reduction in extracellular water and glycosaminglycans content and an increase in collagen type V (Vogel, 1991; Nakagawa et al., 1994; Tuite et al., 1997; Dressler et al., 2002; Kjaer, 2004). These changes may lead to altered biomechanical properties of tendons *in vivo*, which in turn, could affect the overall function of the MTUs. In addition to biological changes, altered environmental mechanical stress may influence ageing tendon. Unloading of the leg extensor MTUs can occur due to immobilization and inactivity, and can lead to muscle atrophy. *In vivo* studies have demonstrated that chronic inactivity (20–90 days bed rest) results in a reduction in tendon stiffness (Kubo et al., 2004; Reeves et al., 2005). Collectively, this means that ageing tendon is affected not only by processes of biological ageing *per se*, but also the reduced habitual loading due to decreased physical activity and muscle strength (Iannuzzi-Sucich et al., 2002; Lauretani et al., 2003).

Since tendon is a mechanosensitive and adaptive tissue, its properties can change depending on its exposure to mechanical loading (Tkaczuk, 1968; Butler et al., 1978; Woo et al., 1980, 1982). Such changes are believed to be regulated through mechanotransduction (Chiquet et al., 2009). Mechanical load generated by the muscle contractions deforms the tendinous tissue, whereby the resultant tendon strain is transferred to its cellular cytoskeleton via the extracellular matrix, causing structural changes (Wang, 2006) and various molecular responses (Robbins and Vogel, 1994; Pins et al., 1997; Arnoczky et al., 2002; Yang et al., 2004; Olesen et al., 2006). These responses have been linked to modifications in tendon mechanical properties following long-term mechanical loading (Kjaer, 2004; Wang, 2006; Heinemeier and Kjaer, 2011; Galloway et al., 2013).

For older adults in particular, the capacities of the leg-extensor MTUs are highly relevant for locomotion. Karamanidis and Arampatzis (2007) and Karamanidis et al. (2008) found significant associations between leg extensor MTU mechanical properties (i.e., TS and QF muscle strength and PT stiffness) and stability control following sudden release from a forward-inclined body position and Onambele et al. (2006) reported that AT stiffness was a decisive predictor of single leg stance ability. Additionally, Stenroth et al. (2015) found that lower AT stiffness was associated with slower timed “up and go” test and 6-min walk test results among healthy older adults. Interventions to counteract age-related changes in the leg extensor MTU mechanical properties may have the potential to positively influence the safety and effectiveness of human locomotion.

This review aims to examine age-related differences in human leg extensor MTU biomechanical properties and if changes in these properties can be counteracted in older adults. Therefore, we provide an overview of recent literature examining age-related differences in human leg extensor MTU biomechanical properties in young and older healthy adults, including muscle strength and the mechanical (tendon stiffness), morphological (tendon cross sectional area: CSA) and material (Young's modulus of the tendon) properties of the AT and PT. Secondly, mechanical loading interventions to trigger alterations in these properties in older adults are reviewed, in order to determine if and to what extent age-related changes can be counteracted and if particular criteria for successful interventions exist.

## Age-related changes in human muscle-tendon unit biomechanical properties

### Muscle strength

**Figure 1:**
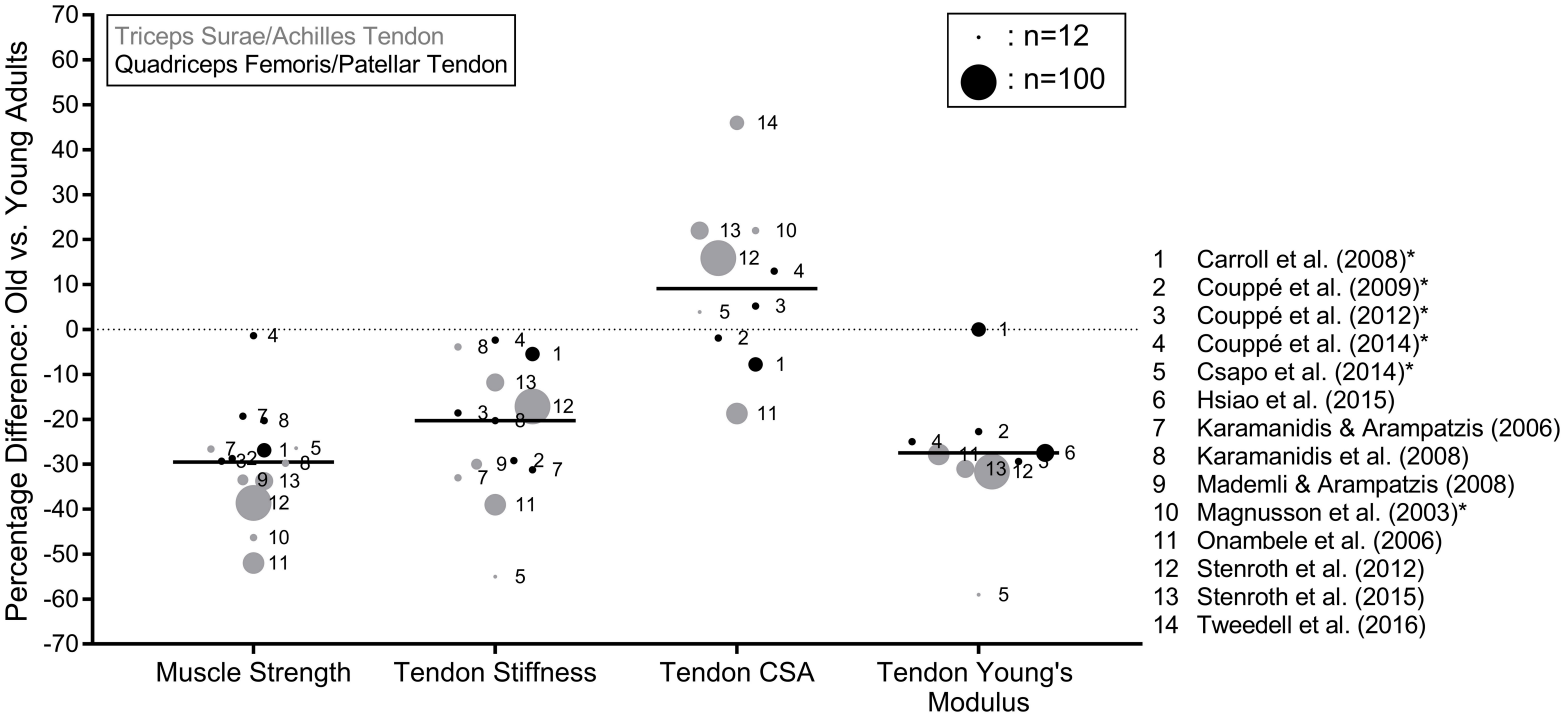
Percentage differences in triceps surae and quadriceps femoris MTU biomechanical properties between older (mean age of 60 years or older) and younger (mean age of 30 years or younger) adults reported in the literature. The black lines represent the median values of the means taken from the studies and the circle size is an approximate representation of the relative sample size. ^*^Indicates the studies that assessed tendon CSA with MRI. Muscle strength was determined during maximum voluntary contractions and reported in the original studies in kg, N, Nm or body weight normalized values.

As the leg extensor MTUs are comprised of muscular and tendinous tissue, any alterations in the tendon biomechanical properties must be interpreted in parallel with changes in the muscle. Twelve articles discussed in this review that examined the tendon biomechanical properties also reported muscle strength [determined during maximum voluntary contractions (MVC) and reported in kg, N, Nm or body weight normalized values]. Seven of the 12 articles analyzed the TS and six assessed the QF MTU. The median number of older adults assessed in the studies was 11 (range of 6–67), with mean ages from the studies ranging from 64 to 79 years. Overall, the age-related changes in muscle strength ranged from −52 to −26.4% for the TS and −29.3 to −1.4% for the QF with an overall median of −29% (Figure 1).

### Tendon stiffness

Tendon stiffness describes the force-elongation relationship of the tendon, assessed in the linear region of the tendon force-elongation relationship. Eleven articles that examined age-related differences in tendon stiffness are discussed in this review (Figure 1). Seven of the articles analyzed the AT and six assessed the PT. The median number of older adults assessed in the studies was 12 (range of 6–67), with mean ages from the studies ranging from 64 to 79 years. Overall, the age-related differences in tendon stiffness ranged from −55 to −3.9% for the AT and −31.2 to −2.4% for the PT with an overall median of −20.3% (Figure 1). One study (Csapo et al., 2014) used combined MRI and dynamometry to assess tendon stiffness, whereas all other articles employed synchronized ultrasonography and dynamometry (e.g., Mademli and Arampatzis, 2008). Despite the large range in percentage differences, which may have been a result of methodological differences affecting the assessment of tendon elongation and stiffness such as imaging method (i.e., Csapo et al., 2014), contraction protocol (Kösters et al., 2014; McCrum et al., 2017) or technical differences between the studies (Finni et al., 2013; Seynnes et al., 2015), the literature shows a consistent reduction in tendon stiffness with age.

### Tendon cross sectional area

Ten of the discussed studies examined tendon CSA, six of which assessed the AT, with the other four analyzing the PT (Figure 1). Four of the six studies that analyzed the AT used ultrasonography (Onambele et al., 2006; Stenroth et al., 2012, 2015; Tweedell et al., 2016), with the other two using MRI (Magnusson et al., 2003; Csapo et al., 2014), whereas all of the studies examining the PT used MRI to assess the CSA (Carroll et al., 2008; Couppé et al., 2009, 2012, 2014). The median number of older adults included in the studies was 15 (range of 6–67), with a range of mean ages from the studies of 65–79 years. Two of the studies found significantly smaller tendon CSA in the older adults (−18.7 and −7.8%; Onambele et al., 2006; Carroll et al., 2008), while four found significantly greater CSA (Magnusson et al., 2003; Stenroth et al., 2012, 2015; Couppé et al., 2014; Tweedell et al., 2016), with an overall median of 9.1% greater tendon CSA in the older adults (Figure 1). As the accuracy of ultrasound-based methods for determining both AT and PT CSA has been shown to be insufficient (Ekizos et al., 2013; Bohm et al., 2016), we suggest that more weight should be given to studies which have used MRI to determine tendon CSA and Young's modulus. If only MRI studies are taken into account, the median difference drops to 4.6%. Aside from imaging methodology, there is variation in how CSA was determined. Most AT studies assessed CSA at a specific tendon length (usually where the CSA is assumed to be smallest), which varied between three and four cm proximal to the insertion of the AT to the calcaneus (Magnusson et al., 2003; Stenroth et al., 2012, 2015; Csapo et al., 2014). The remaining AT and PT studies used multiple (usually three) lengths from which the CSA was averaged. As a result, potential region-specific differences in the CSA between younger and older adults may be excluded, as no existing study has compared the AT or PT CSA between older and younger adults along the entire tendon length; a potentially important gap in the literature. Training-induced regional changes have previously been reported in young adults (Magnusson and Kjaer, 2003; Arampatzis et al., 2007; Kongsgaard et al., 2007; Seynnes et al., 2009). Due to the diversity in methodologies and results, no firm conclusion can be made about the age-effects on tendon CSA.

### Tendon young's modulus

The Young's modulus of a material is defined as the slope of the stress-strain relationship, where stress is tendon force relative to CSA and strain is tendon elongation in relation to resting length. Nine of the discussed studies assessed the tendon Young's modulus, with four and five studies analyzing the AT and PT, respectively. The median number of older adults included was 19 (range of 6–67), with a range of mean ages from 64.5 to 76.7 years. One of the four studies of the AT (Csapo et al., 2014) and all but one (Hsiao et al., 2015) of the five studies of the PT used MRI to assess the tendon CSA, with the others using ultrasound to assess tendon CSA. All but one of the studies used synchronized dynamometry and ultrasound to assess the force-elongation behavior of the tendon, with the final study (Csapo et al., 2014) using combined MRI and dynamometry. A median difference of −27.8% in Young's modulus (−23.9% when only including the MRI-based studies) was found, with no studies showing a higher Young's modulus in older, compared to younger adults (Figure 1). There is a relatively consistent reduction in Young's modulus with age, although the above described limitations regarding region-specific CSA should also be kept in mind. Thus, we can conclude that the observed changes in tendon stiffness due to ageing are predominantly due to changes in tendon material properties rather than reduced CSA.

## Effects of increased mechanical loading on muscle-tendon unit biomechanical properties in older adults

**Figure 2:**
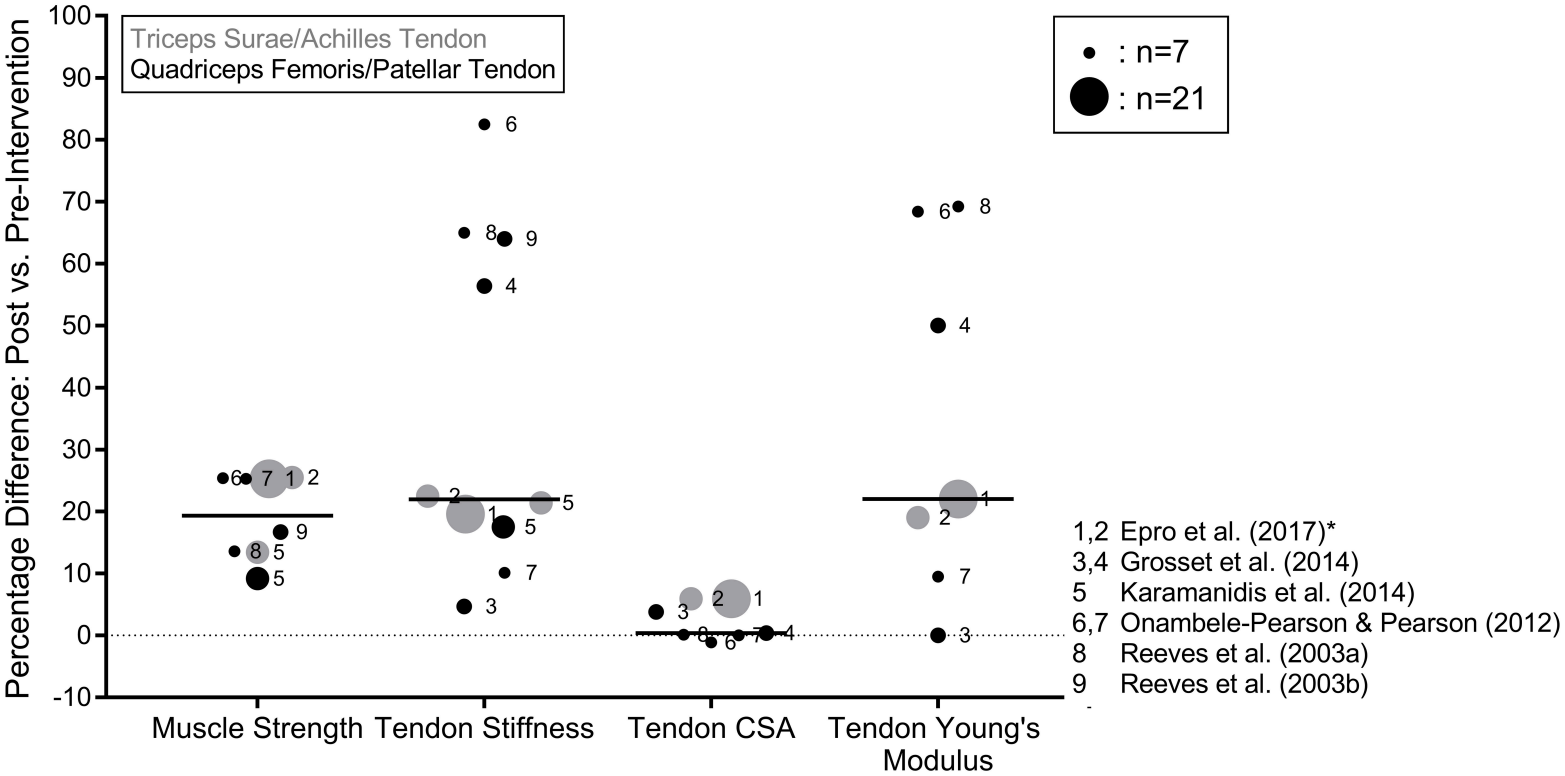
Percentage differences in triceps surae and quadriceps femoris MTU biomechanical properties between pre and post-intervention assessments with older adults. The black lines represent the median values of the means taken from the studies and the circle size is an approximate representation of the relative sample size. Muscle strength was determined during maximum voluntary contractions and reported in the original studies in kg, N, Nm or body weight normalized values. ^*^Indicates the studies that assessed tendon CSA with MRI. Where an article applied more than one intervention, the data were split so that each circle represents the results of the individual interventions within the studies.

In this section, we provide an overview and discussion of intervention studies conducted with older adults (mean age of 60 years or older) that analyzed the leg extensor MTUs' biomechanical properties. Nine intervention groups from six articles are discussed (Figure 2; Reeves et al., 2003a,b; Onambele-Pearson and Pearson, 2012; Grosset et al., 2014; Karamanidis et al., 2014; Epro et al., 2017). All interventions consisted of predominantly resistance-based exercise and lasted 12–14 weeks in length, with one study also conducting a 1.5 year long intervention (Epro et al., 2017). All conducted two or three sessions per week that were partly or completely supervised. The exercise protocols ranged from highly specific and controlled protocols (i.e., five sets and four repetitions of isometric plantar flexions at 90% MVC held for 3 s guided by visual feedback in Epro et al., 2017) to more mixed ecological training interventions with multiple strength exercises, as well as hopping or running (Onambele-Pearson and Pearson, 2012; Grosset et al., 2014; Karamanidis et al., 2014). The number of contractions per exercise ranged from 16 to 44 spread over a range of two to five sets, and all but one of the interventions in the study of Grosset et al. (2014) aimed to impose high mechanical loads (e.g., 80–90% MVC or 80% of five repetition maximum for 10 repetitions). Grosset et al. (2014) compared low and high intensity training groups (40% vs. 80% MVC) and Onambele-Pearson and Pearson (2012) compared male and female groups of older adults. One article (two intervention durations) focused exclusively on the AT (Epro et al., 2017), four articles (six intervention groups) focused exclusively on the PT (Reeves et al., 2003a,b; Onambele-Pearson and Pearson, 2012; Grosset et al., 2014) and one article conducted an intervention targeting both the TS and QF MTUs (Karamanidis et al., 2014). One study did not report muscle strength values (Grosset et al., 2014) but all other articles reported significant muscle strength increases (Figure 2) (13.4–25.5% for the TS and 9.2–25.4% for the QF), measured either by maximum joint moments, maximum force during isometric contractions or by one or five repetition maximum values (Reeves et al., 2003a,b; Onambele-Pearson and Pearson, 2012; Karamanidis et al., 2014; Epro et al., 2017). All but one intervention resulted in significant increases in AT (19.6–22.5%) or PT (10.1–82.5%) stiffness (Figure 2), with the one non-significant result coming from the low intensity (40% MVC) training group of Grosset et al. (2014). Young's modulus of the tendons was assessed by four of the articles, generally showing significant increases in both the AT (19–22%) and PT (9.5–68.4%), with the low intensity group of Grosset et al. (2014) showing no change (Figure 2). Tendon CSA was also assessed by four of the articles, three of which assessed the PT and found no differences post-intervention (Reeves et al., 2003a; Onambele-Pearson and Pearson, 2012; Grosset et al., 2014) and one reported significant increases in AT CSA after both 14 weeks and 1.5 years of intervention (Epro et al., 2017). It is noteworthy that Epro et al. (2017) analyzed the CSA over the entire length of the AT using MRI, whereas the other studies used ultrasound and did not assess the entire length of the AT. This may suggest that the ultrasound method is not sensitive enough to consistently detect the usual range of changes in tendon CSA following exercise interventions (a range of 3.7–9.6% from the studies using MRI reported in the review of Bohm et al., 2015 and in Epro et al., 2017).

Older tendons appear to preserve their adaptability to mechanical loading with age (Reeves et al., 2003a,b; Onambele-Pearson and Pearson, 2012; Grosset et al., 2014; Karamanidis et al., 2014; Epro et al., 2017), but a few results are worth noting when considering the effectiveness of the interventions. Firstly, Epro et al. (2017) found that 14 weeks of resistance exercise was a sufficient time period to trigger adaptive changes in the biomechanical properties of the AT and that these adaptations were maintained for 1.5 years by continuing training, suggesting that there is a non-linear time-response relationship of ageing tendons subjected to mechanical loading. However, the lack of further adaptation may have been related to a plateau in plantarflexion MVC force after 11–12 weeks of training (Epro et al., 2017). These long-term adaptation processes should be investigated in future research. Secondly, the intervention with the lowest exercise intensity (Grosset et al., 2014) and therefore, lowest tendon strain magnitudes, was the only intervention that found no significant changes in the tendon biomechanical properties. This finding is in accordance with evidence from young adults, demonstrating that tendon adaptation is triggered only when a specific threshold of strain magnitude is exceeded during the loading exercise (Arampatzis et al., 2007; Bohm et al., 2015). This might also explain the absence of differences in lower limb MTU biomechanical properties in a number of cross-sectional studies of older endurance runners and their age-matched sedentary counterparts (Karamanidis and Arampatzis, 2005, 2006). Future research should continue to explore viable activities for stimulating tendon adaptation in older adults.

In young adults, studies have reported increased tendon stiffness, CSA and Young's modulus in response to tendon loading exercise over 12–14 weeks (Kubo et al., 2001; Arampatzis et al., 2007; Kongsgaard et al., 2007; Bohm et al., 2015; Wiesinger et al., 2015). The importance of strain magnitude for tendon adaptation was originally demonstrated by Arampatzis et al. (2007) and two systematic reviews concluded that resistance training can lead to tendon adaptation providing that sufficient tendon strain magnitudes (or intensities greater than 70% MVC) are applied (Bohm et al., 2015; Wiesinger et al., 2015). This is in agreement with the results found in the current review in the study of Grosset et al. (2014). Considering the results in older adults, the adaptation magnitudes in stiffness and Young's modulus are similar to those observed after 12–14 weeks exercise in younger adults (increases of 16–36% and 15–45% respectively; Arampatzis et al., 2007, 2010; Kubo et al., 2007; Fletcher et al., 2010; Fouré et al., 2013; Bohm et al., 2014). Moreover, the changes in tendon CSA values after exercise are consistent with younger adults (mean AT CSA increases of between 0.5 and 10%; Arampatzis et al., 2007, 2010; Kongsgaard et al., 2007; Bohm et al., 2014). Importantly, the cyclic tendon strain exercise protocol of Epro et al. (2017) in older adults was the same as Arampatzis et al. (2007) in young adults and relative adaptations in the TS MTU biomechanical properties were similar. Overall, it appears that the leg extensor MTUs of older adults respond to increased mechanical loading in a way that involves similar magnitudes of tendon and muscle adaptation.

## Conclusion

Based on the available literature, increasing age appears to result in reductions in human TS and QF muscle strength accompanied by reductions in AT and PT stiffness and elastic modulus, whereas the effect on AT and PT CSA is unclear. Therefore, the observed changes in tendon stiffness due to ageing are predominantly due to changes in tendon material properties rather than changes in tendon CSA. However, tendons appear to retain their mechanosensitivity with age, showing similar alterations in their biomechanical properties in older adults compared to younger adults following training interventions. Exercise interventions should implement tendon strains corresponding to high, repetitive mechanical loading (i.e., 80–90% of MVC) for up to 3 or 4 months in order to successfully counteract age-related changes in leg extensor MTU biomechanical properties.

## Author contributions

Conception of the work: CM and PL; literature acquisition: MK, GE, PL, and CM; literature synthesis: PL, CM, and MK; analysis and interpretation: all authors; drafted the manuscript: CM; prepared figures: CM, PL, and MK; revised the manuscript for important intellectual content: all authors; final approval of the version to be published: all authors; agreement to be accountable for the work: all authors.

### Conflict of interest statement

The authors declare that the research was conducted in the absence of any commercial or financial relationships that could be construed as a potential conflict of interest.
